# Pyomyoma in a primigravida at term: a case report

**DOI:** 10.11604/pamj.2022.41.180.33913

**Published:** 2022-03-07

**Authors:** Uchechukwu George Ogbu, Abayomi Ibukun Alao, Uche Augustine Akunaeziri, Reuben Chido-Abba

**Affiliations:** 1 Department of Obstetrics and Gynaecology, Federal Medical Centre, Keffi, Nasarawa State, Nigeria,; 2 Westcare Hospital, Ejigbo, Lagos State, Nigeria

**Keywords:** Pyomyoma, leiomyoma, primigravida, case report

## Abstract

Pyomyoma is a rare complication of uterine fibroid occurring most commonly in pregnancy, post-partum, post-abortion, and post-menopausal periods. It results from infarction, necrosis and secondary infection of leiomyoma. We report a case of 29-year-old primigravida with breech at term co-existing with cystic degenerative uterine fibroid, who presented with recurrent fever and abdominal pain. She had caesarean section and was found to have pyomyoma intraoperatively. She subsequently had drainage of pus, caesarean myomectomy and antibiotics therapy. She had good outcome and was discharged on fifth postoperative day. Pyomyoma should be suspected in pregnant women with leiomyoma, unexplained recurrent fever, abdominal pain and cystic degenerative changes on ultrasound.

## Introduction

Uterine fibroid (myoma or leiomyoma) is the commonest benign tumor of uterine smooth muscle [1,2]. Incidence of 20-40% have been reported in women of the reproductive age group [1]. Pyomyoma or suppurative leiomyoma is a rare life threatening complication of uterine fibroid [1-4]. It results from infarction, necrosis and secondary infection of leiomyoma [3,4]. Pyomyoma have been reported in pregnancy, postpartum, post-abortion and in postmenopausal women [2-10].

The clinical presentation such as abdominal pain, fever is not specific and often confused with symptoms of fibroid degeneration [7]. Diagnosis of pyomyoma is challenging because it is rare, presents with non-classical symptoms and may take a long time to develop, leading to delay in diagnosis [2-10]. Due to the late diagnosis, it is often associated with increased morbidity and mortality [2-8]. Ultrasound diagnosis is difficult due to paucity of reported imaging and difficulty in differentiating it from degenerated fibroid [8]. Despite the high prevalence of uterine fibroid in sub-Saharan Africa, only few cases have been reported. We report a case of pyomyoma in a primigravida with breech presentation at term. She had caesarean myomectomy with good outcome.

## Patient and observation

**Patient information:** she was a 29-year-old primigravida who presented to the obstetrics emergency unit at a gestational age of 39 weeks + 1 day with complaint of abdominal pain and fever of 24 hours duration. She booked for antenatal care at gestational age of 9 weeks + 4 days. Obstetric ultrasound scan done at booking diagnosed a viable pregnancy co-existing with uterine fibroid. She had repeated episodes of fever and abdominal pain during pregnancy. She was admitted on several occasions and managed for suspected red degeneration of fibroid in pregnancy at 19 weeks gestational age, malaria in pregnancy, anaemia in pregnancy at 27 weeks gestational age and preterm labour at 36 weeks gestational age. There was history of symptomatic uterine fibroid in her elder sister and she had abdominal myomectomy.

**Clinical findings:** on physical examination, she was in painful distress, not pale, anicteric, not cyanosed, febrile with a temperature of 38.1°C. She was not dehydrated and had bilateral pedal oedema. Her respiratory rate was 28 cycles/minute, with vesicular breath sounds. The pulse was 98 beats per minute and blood pressure was 110/70 mmHg. The abdomen was enlarged with epigastric and umbilical tenderness. The liver, spleen and kidneys were not palpably enlarged. The symphio-fundal height was 43 cm which was large for date. A singleton fetus in longitudinal lie, breech presentation was palpated. She had no contraction in 10 minutes and the fetal heart tone was 126 beats/minute and it was regular. Pelvic examination showed that the cervix was soft, 3cm in length and cervical os was closed. There were no adnexal tenderness or mass bilaterally.

**Diagnostic assessment:** the full blood count done showed a packed cell volume of 32%, a white cell count of 16 x 10^9^/L, platelet count of 175,000/ml. Urinalysis was normal, urine culture yielded no growth after 48 hours of incubation. C-reactive protein was elevated with value of 94 mg/l. Malaria parasite was negative. The serum electrolyte, urea and creatinine were within normal range. HIV, HBsAg and HCV screening were negative. Abdominopelvic ultrasound done showed single intrauterine breech presenting fetus with good cardiac activity at 38 weeks + 5 days co-existing with a solitary fundal sited cystic degenerating uterine fibroid measuring about 12.5 x 7.8cm. The other intra-abdominal organs were sonographically normal.

**Diagnosis:** a diagnosis of breech presentation at term in a primigravida with co-existing cystic degenerating uterine fibroid was made.

**Figure 1:**
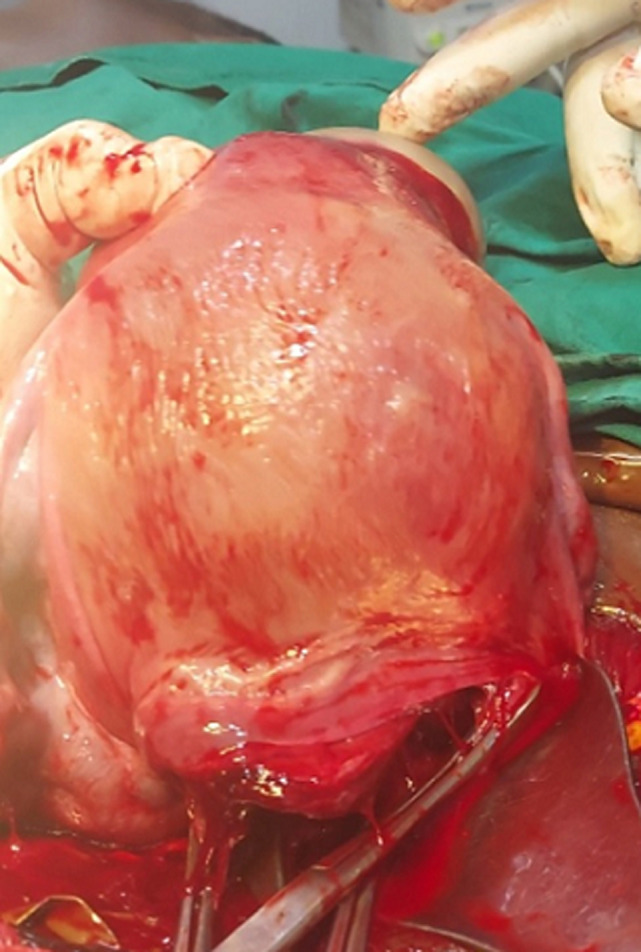
the uterus following the delivery of the baby

**Figure 2:**
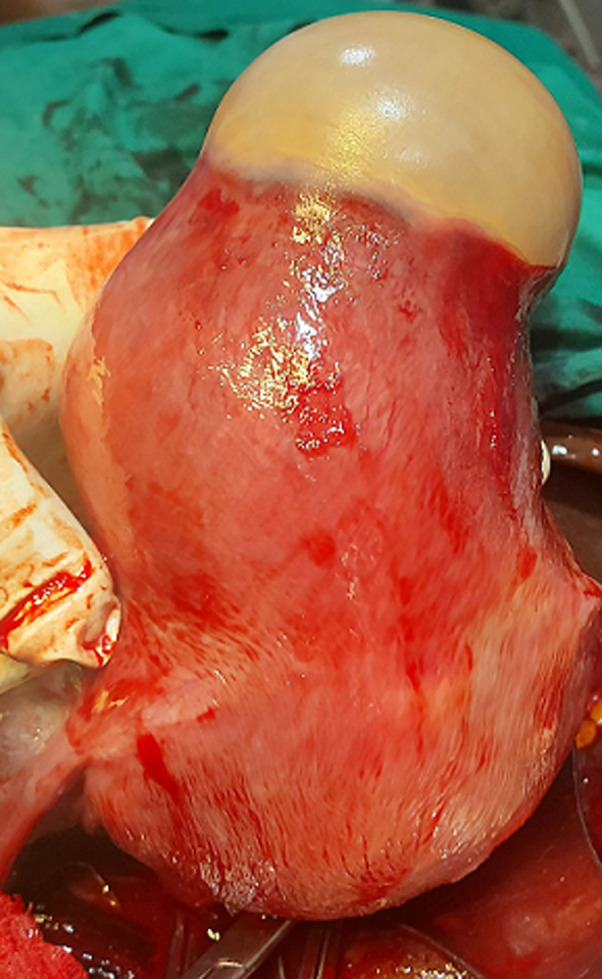
the anatomical location of the pyomyoma

**Figure 3:**
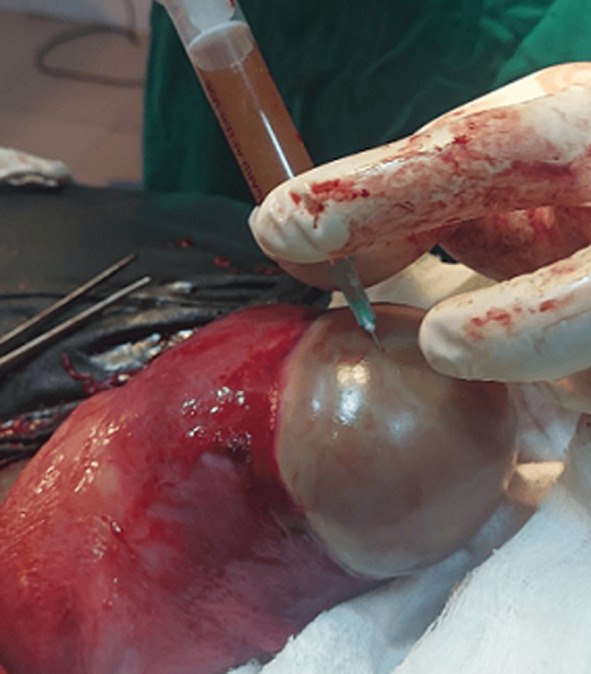
aspiration of pus for microscopy, culture and sensitivity

**Figure 4:**
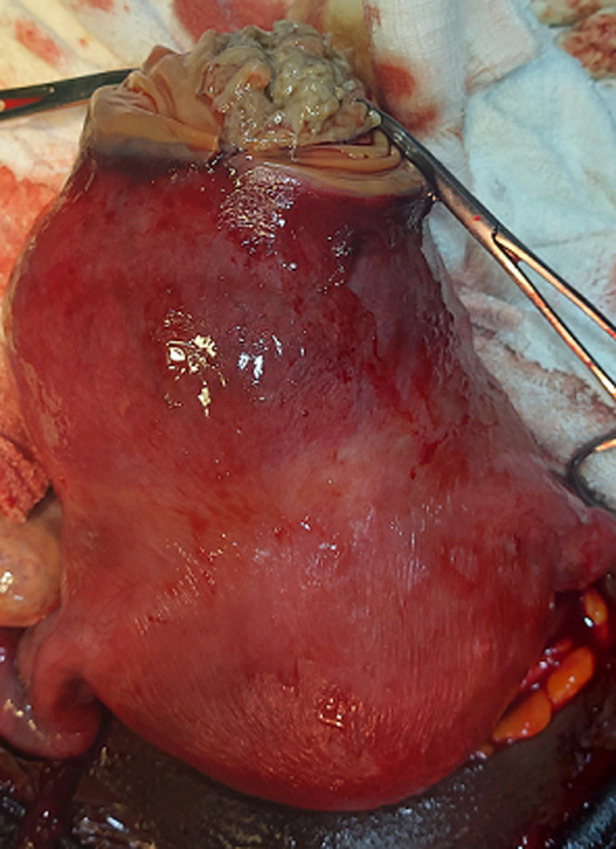
suppuration in the myoma with surrounding areas of hyperemia

**Figure 5:**
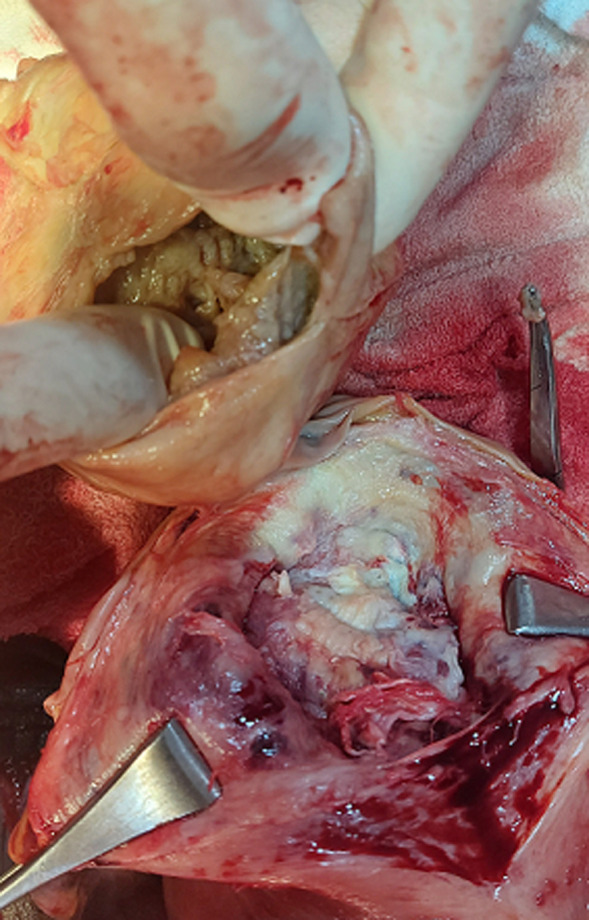
cavity left after the removal of the pyomyoma

**Therapeutic interventions:** she received intramuscular paracetamol 900mg and pentazocine 30mg stat, intravenous ceftriaxone acid 1g 12-hourly and intravenous metronidazole 500mg 12-hourly for 72-hours. She was counseled on caesarean section and she gave a written consent. She subsequently had caesarean section at 39 weeks + 4 days. Intraoperative findings were alive male neonate in breech presentation with Apgar score of 8 and 9 in 1^st^ and 5^th^ minute respectively, with birth weight of 3.52kg, a posterior-fundal sited subserous myoma measuring about 12cm x 10cm which contained 60mls of straw to gray coloured purulent fluid and surrounding areas of hyperemia (Figure 1, Figure 2, Figure 3, Figure 4, Figure 5). The purulent fluid was aspirated and sent for microscopy, culture and sensitivity. There was also multiple tiny subserous myoma on the anterior and posterior uterine wall. The anterior lower segment uterine incision was closed in two layers with vicryl 1 suture. A haemostatic tourniquet using a foley´s cathater size 20F was then applied at the isthmus and caesarean myomectomy performed. Figure 5 depicts the cavity left following the removal of the pyomyoma. The dead space left following the removal of the pyomyoma was closed in layers using vicryl 1 suture. Adequate haemostatis were ensured. The peritoneal cavity was irrigated with 1.5 litres of warm normal saline and suctioned. The uterus was returned into the peritoneal cavity and anterior abdominal wall closed in layers. The specimen was sent for histology. She was placed on intravenous ceftriaxone and metronidazole for 72 hours, intravenous fluids and analgesia. Her vital signs were normal postoperative. Postoperative packed cell volume was 30%, pus culture yielded heavy growth of *Escherichia coli* which was sensitive to ceftriaxone, cefixime, amoxicillin and ofloxacin. She was placed on oral cefixime 200mg BD and metronidazole 400mg TDS for 5 days after 72 hours of parenteral antibiotics. She made satisfactory recovery and was discharged home on fifth postoperative day.

**Follow-up and outcome of interventions:** she had two satisfactory postnatal clinic visits and she had no complaints. Histology report showed a degenerating leiomyoma with areas of cystic degeneration, necrosis, foci of suppuration and inflammatory cells infiltrations. There was no neoplastic changes. She was counselled on the possibility of recurrence.

**Patient perspective:** she was satisfied with her management. She informed us that she no longer experiences persistent fever and abdominal pain.

**Informed consent:** her permission for this case to be reported was sort and she gave a written consent.

## Discussion

Pyomyoma is a rare complication of leiomyoma that was first reported in 1871 and about 100 cases of pyomyoma have been reported since then [5,10]. Despite leiomyoma being commoner among blacks, only two cases have been reported in sub-Saharan Africa [6]. In most of the reported cases, pyomyoma occurred in women in first and second trimester, puerperium, post-abortion, following uterine instrumentation and uterine artery embolization [2-10]. It has also been reported in postmenopausal and immunosuppressed women [2,9]. There was no previously reported case of pyomyoma in a term pregnancy in the literatures reviewed like in this index case. Although there are several hypotheses, the definitive cause of a pyomyoma remains unclear [9].

Pyomyoma was postulated to result from bacterial infection of necrotic/ishaemic foci [2-10]. This maybe as a result of vascular compromise in postmenopausal women or haemorrhage and necrosis during pregnancy [7,9]. The bacteria reach the necrotic foci from the uterine cavity and/or adjacent organs like the bowels and adnexa or by haematogenous and lymphatic spread [2-9]. The common organisms found in pyomyoma include *Staphylococcus aureus, Streptococcus haemolyticus, Proteus, Klebsiella spp, Streptococcus agalactiae, E. coli, Enterococcus faecalis*, and *Sphingomonas paucimobilis* [2-10]. Pus collected from our patient cultured heavy growth of *E. coli*.

Pyomyoma has non-specific presentations, thus making its diagnosis difficult [2-10]. It presents with non-classical symptoms such as fever and abdominal pain which may take a long time to develop, leading to delay in diagnosis [2-10]. Pyomyoma may be clinically silent or subacute despite producing continuous bacteremia and this makes the diagnosis difficult [2-10]. This index case presented with fever and abdominal pain. Delay in diagnosis may lead to rupture of pyomyoma and peritonitis [5,6,8]. These patients with ruptured pyomyoma in reported cases, presented with acute abdomen and sepsis [5,6,8].

Ultrasonography and Computerized Tomography (CT) scan are imaging techniques that aid in diagnosis of pyomyoma. Sonographic features suggestive of pyomyoma include a heterogenous mass with cystic and solid components however, these features are non-specific [2,6,8]. These sonographic findings could also be seen in uterine fibroids with cystic changes. CT scan is a better imagining technique and often reveals a heterogeneous pelvic mass with gas and debris [2,8]. Presence of gas in a myoma is highly suggestive of pyomyoma on CT scan [2,6]. This was not done for this patient because of cost and controversies surrounding the safety of CT scan in pregnancy.

The definitive treatment of pyomyoma involves antibiotics therapy, myomectomy or hysterectomy [2,3,6,9,10]. Myomectomy is the surgical option of choice in patients that desire future fertility [2,3,6,9,10]. Our patient had caesarean myomectomy and antibiotics therapy because she was a primigravida who desired future fertility.

Complications of pyomyoma include uterine rupture, peritonitis, overwhelming sepsis and death [9,10]. The mortality associated with pyomyoma is high especially if there is rapid progression, delay in diagnosis and definitive treatment [9,10]. Therefore, only medical treatments are not encouraged [9,10]. Case fatalities were reported among immunosuppressed patients and intravenous drug users in some of the previous case reports [5,8,9].

This case was reported because pyomyoma is rare and there is no reported case of pyomyoma co-existing with term pregnancy in literatures reviewed. Clinicians should have high index of suspicion when managing patients with leiomyoma in pregnancy presenting with persistent fever, abdominal pain and preterm labour.

## Conclusion

Pyomyoma in pregnancy is a rare complication of uterine fibroid and should be considered in patients with persistent unexplained fever, abdominal pain and leiomyoma with cystic degenerative changes on ultrasound. Myomectomy and antibiotics are vital in achieving good outcome in patients that desire future fertility.
